# Identification of Molecular Characteristics and New Prognostic Targets for Thymoma by Multiomics Analysis

**DOI:** 10.1155/2021/5587441

**Published:** 2021-05-19

**Authors:** Dazhong Liu, Pengfei Zhang, Jiaying Zhao, Lei Yang, Wei Wang

**Affiliations:** Department of Thoracic Surgery, Second Affiliated Hospital of Harbin Medical University, Harbin 150086, China

## Abstract

**Background:**

Thymoma is a heterogeneous tumor originated from thymic epithelial cells. The molecular mechanism of thymoma remains unclear.

**Methods:**

The expression profile, methylation, and mutation data of thymoma were obtained from TCGA database. The coexpression network was constructed using the variance of gene expression through WGCNA. Enrichment analysis using clusterProfiler R package and overall survival (OS) analysis by Kaplan-Meier method were carried out for the intersection of differential expression genes (DEGs) screened by limma R package and important module genes. PPI network was constructed based on STRING database for genes with significant impact on survival. The impact of key genes on the prognosis of thymoma was evaluated by ROC curve and Cox regression model. Finally, the immune cell infiltration, methylation modification, and gene mutation were calculated.

**Results:**

We obtained eleven coexpression modules, and three of them were higher positively correlated with thymoma. DEGs in these three modules mainly involved in MAPK cascade and PPAR pathway. LIPE, MYH6, ACTG2, KLF4, SULT4A1, and TF were identified as key genes through the PPI network. AUC values of LIPE were the highest. Cox regression analysis showed that low expression of LIPE was a prognostic risk factor for thymoma. In addition, there was a high correlation between LIPE and T cells. Importantly, the expression of LIPE was modified by methylation. Among all the mutated genes, GTF2I had the highest mutation frequency.

**Conclusion:**

These results suggested that the molecular mechanism of thymoma may be related to immune inflammation. LIPE may be the key genes affecting prognosis of thymoma. Our findings will help to elucidate the pathogenesis and therapeutic targets of thymoma.

## 1. Introduction

Thymoma is the most common anterior mediastinal compartment tumor, originating from the thymic epithelial cell population [1]. The incidence of thymomas is approximately 2.5 cases per million people per year, with an age distribution ranging from 10 to 80 years [2]. In addition, thymoma is often associated with autoimmune diseases, especially myasthenia gravis (MG) [3, 4]. However, the potential molecular oncogenesis of thymoma remains unknown. Generally, when a thymoma is diagnosed, the patient will receive surgical treatment. For stages III and IV patients, the 5-year survival rates were 74% and <25%, respectively [5]. At present, neither surgeon nor physician can predict the prognosis and metastasis status of thymoma patients through X-ray examination, nor can detailed treatment plan be formulated before operation [6]. Obviously, the establishment of additional predictors is very beneficial for the identification and treatment of thymoma.

The pathogenesis of thymoma is various, and the rapid development of “genome” technology, including whole-genome expression analysis and next-generation sequencing (NGS), provides new means to explore the complexity and map of genomic alterations in thymoma [7–9]. Epigenetic modifications, including epigenetic alterations, are a feature of cancer because they play an important role in the process of carcinogenesis [10, 11]. In addition, the thymus provides a special microenvironment for the development and selection of mature T cells. Recent evidence suggests that immune responses such as T cells are involved in the development of thymoma [12, 13]. However, the understanding of the pathogenesis of thymoma is still limited.

In recent years, with the development of molecular biology, more and more research projects have begun to explore methods to accurately predict the prognosis of thymoma. In this study, we used multiomics datasets from the tumor genome map (TCGA). The results may be helpful to understand the pathogenesis of thymoma and identify LIPE as a potential new therapeutic target through bioinformatics analysis. The novelty of this work is that we combined the variance and difference of gene expression to screen the genes related to the prognosis of thymoma through coexpression network and PPI network. Then, the key genes were further screened by methylation modification.

## 2. Materials and Methods

### 2.1. TCGA Dataset Processing and Coexpression Analysis

Thymoma mRNA-seq expression data, methylation data, mutation data, and clinical materials were obtained from TCGA website (https://portal.gdc.cancer.gov/). The variance of gene expression was calculated, and the top 1/4 genes were intercepted for coexpression analysis through weighted gene coexpression network analysis (WGCNA).

### 2.2. Screening of Differentially Expressed Genes

The differentially expressed genes (DEGs) between thymoma and control were identified by limma R package. Set the filtering threshold *P* < 0.05.

### 2.3. Construction of PPI Network

The gene was mapped into the STRING database (https://string-db.org) to obtain the protein-protein interaction (PPI) network. A significant PPI network was obtained by comprehensive score ≥ 0.7, which was demonstrated by the Cytoscape software. The selection of key genes was based on their association with other proteins: genes with higher connectivity were considered to play an important role in the PPI network [14, 15].

### 2.4. Enrichment Analysis

In order to analyze the biological functions and signaling pathways of differentially expressed genes in thymoma-related modules, we performed enrichment analysis. Gene Ontology (GO) and the Kyoto Encyclopaedia of Genes and Genomes (KEGG) were enriched by clusterProfiler R package. *P* < 0.05 was the threshold used for the significant terms. Gene set enrichment analysis (GSEA) was performed with the GSEA software for genes [16, 17].

### 2.5. Differential Methylation and Mutation Analysis

The quality of the original probe data obtained from the methylated microarray was checked, including background correction, probe type difference adjustment, and probe exclusion. According to these in sample standardized procedures, DNA methylation was scored as a *β* value. We used samr R package for differential methylation analysis. For a CpG site to be considered differentially methylated, the difference in the median *β* value in thymoma and normal samples should be at least 0.1 and the *P* value <0.05. The nonsilent mutation (gene-level) data were analyzed using Maftools R-package.

### 2.6. Statistical Analysis

Statistical analysis was performed using the SPSS software, version 23.0 (SPSS Inc., Chicago, USA). Kaplan-Meier method was used to estimate the overall survival (OS). Cox regression model and Cox proportional hazards regression method were used to identify predictors of OS [18]. *P* value <0.05 was considered statistically significant [19].

## 3. Results

### 3.1. Coexpression of Genes in Thymoma

**Figure 1:**
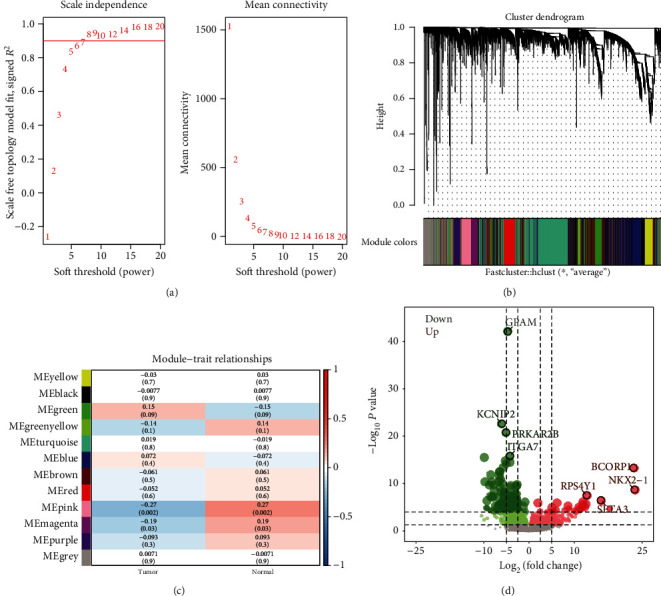
Coexpression analysis of gene expression in thymoma. (a) Determination of soft threshold power in coexpression analysis. The left image shows the scale-free fit index (*y*-axis) as a function of the soft-thresholding power (*x*-axis). The right image shows the average connectivity (degree, *y*-axis) as a function of the soft-thresholding power (*x*-axis). (b) Module cluster tree of thymoma genes with large variance. Branches with different colors correspond to different modules. (c) The correlation between module and clinical trait. Each row corresponds to a module, and each column corresponds to a feature. Each cell contains the corresponding correlation and *P* value. (d) The differentially expressed genes between thymoma and control. Red nodes were significantly upregulated genes, and green nodes were significantly downregulated genes.

According to the variance results of thymoma gene expression, the top 1/4 genes with larger variance were selected for coexpression analysis. A coexpression network consisting of 5758 genes was obtained. Taken 0.9 as the threshold of correlation coefficient, select the soft threshold as 7 (Figure 1(a)). A total of 11 coexpression modules were identified through WGCNA analysis (Figure 1(b)). In addition, we calculated the correlation between module genes and thymoma. We found that MEgreen, MEblue, and MEturquoise had the highest correlation with tumor samples (Figure 1(c)). Furthermore, 2559 differentially expressed genes (DEGs) were screened between thymoma and control group (*P* < 0.05) (Figure 1(d)).

### 3.2. Enrichment of Differentially Expressed Genes in Modules

**Figure 2:**
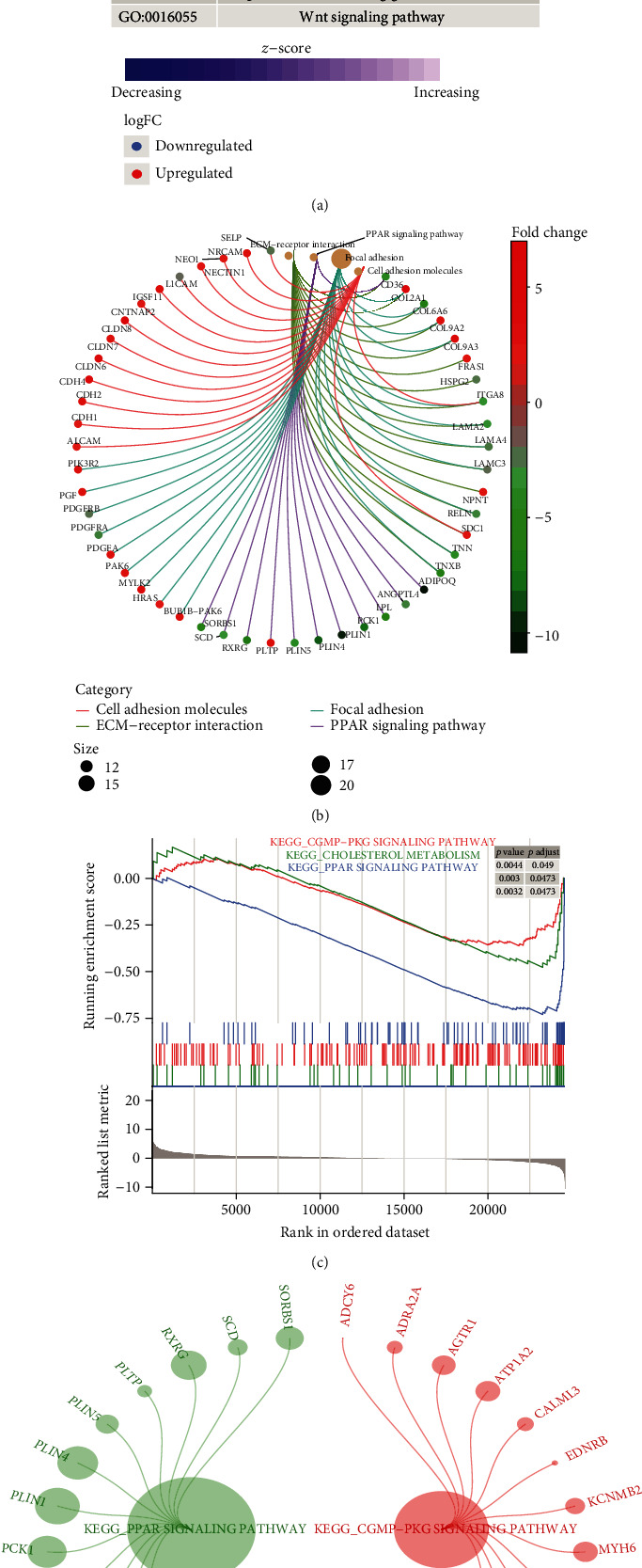
Enrichment analysis of thymoma-related module genes. (a) Important genes were involved in biological processes. Red nodes are upregulated genes, and blue nodes are downregulated genes. (b) Important genes were involved in KEGG pathway. Different line colors represent different signaling pathways which genes involved in. (c) KEGG pathway in GSEA for important genes. These pathways were significantly upregulated in thymoma. (d) The DEGs involved in the same KEGG pathway in the results of enrichment and GSEA. Different colors represent genes involved in different signaling pathways.

Further, 913 intersection genes between DEGs and the three modules with the highest correlation were selected as the important genes for subsequent study and enrichment analysis. The results of GO enrichment showed that these genes were involved in 1234 biological processes (BP), 151 cell components (CC), and 214 molecular functions (MF). It mainly included cell growth, positive regulation of MAPK cascade, ERK1 and ERK2 cascade, response to transforming growth factor beta, and Wnt signaling pathway (Figure 2(a)). KEGG enrichment results showed a total of 40 terms, mainly involving cell adhesion molecules, ECM-receptor interaction, focal adhesion, and PPAR signaling pathway (Figure 2(b)). In addition, the GSEA results showed some of the same results as KEGG, mainly including cGMP-PKG signaling pathway, cholesterol metadata, and PPAR signaling pathway (Figure 2(c)). These same signaling pathways cover a large number of differentially expressed genes (Figure 2(d)).

### 3.3. Identification of Key Prognostic Genes

**Figure 3:**
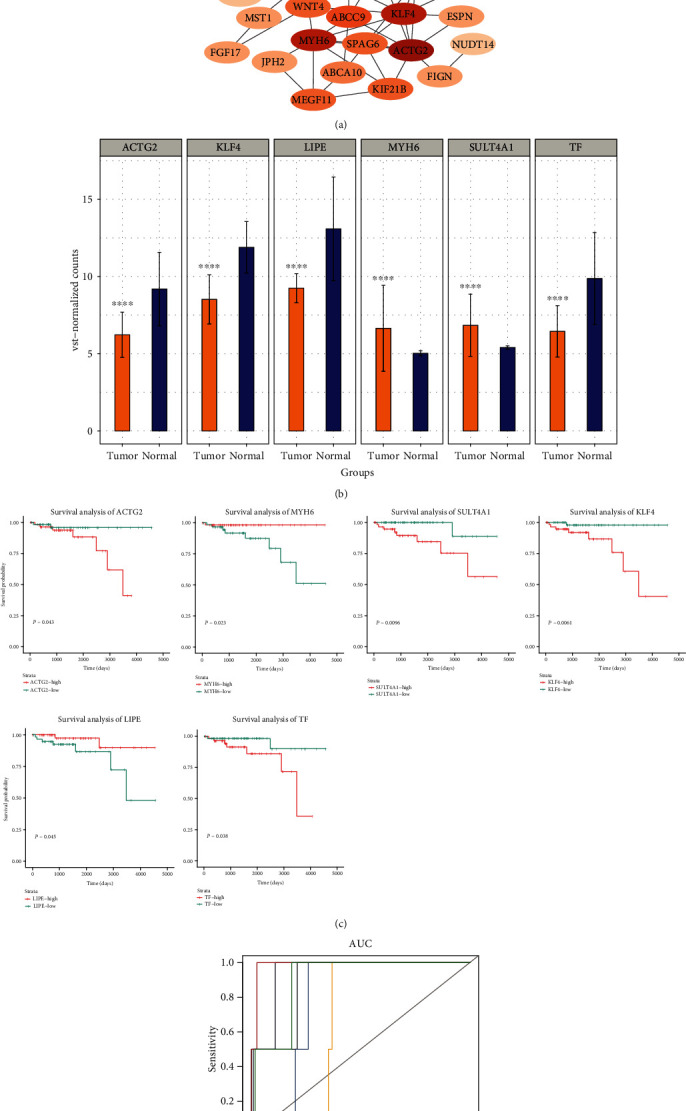
Identification of key genes affecting overall survival of thymoma. (a) Cytoscape software shows the PPI network of important genes based on the STRING database. (b) The expression of six genes with the highest connectivity in the PPI network. ^∗∗∗^*P* < 0.001. (c) The effect of six genes with the highest connectivity in the PPI network on the overall survival of thymoma (Kaplan-Meier plot). Red and green curves are for high expression and low expression, respectively. (d) ROC curve of key genes. Different color curves represent different genes.

The overall survival (OS) analysis of selected important genes identified 88 genes with significant impact on prognosis (*P* < 0.05). Mapping these genes into the STRING database yielded a PPI network of 45 genes, which was displayed by Cytoscape (Figure 3(a)). The top six genes with the highest connectivity were analyzed in depth as key genes. Among them, the expression of MYH6 and SULT4A1 in osteosarcoma was higher than that in control group, while the expression of LIPE, ACTG2, KLF4, and TF was decreased (Figure 3(b)). In addition, high expression of LIPE and MYH6 could improve the OS of patients, and ACTG2, KLF4, SULT4A1, and TF decreased the OS of patients (Figure 3(c)). ROC curves showed that the AUC values of these six genes were all greater than 0.6, especially those of LIPE, and KLF4 and TF were greater than 0.9 (Figure 3(d)).

### 3.4. The Effect of Key Genes on Prognosis

**Figure 4:**
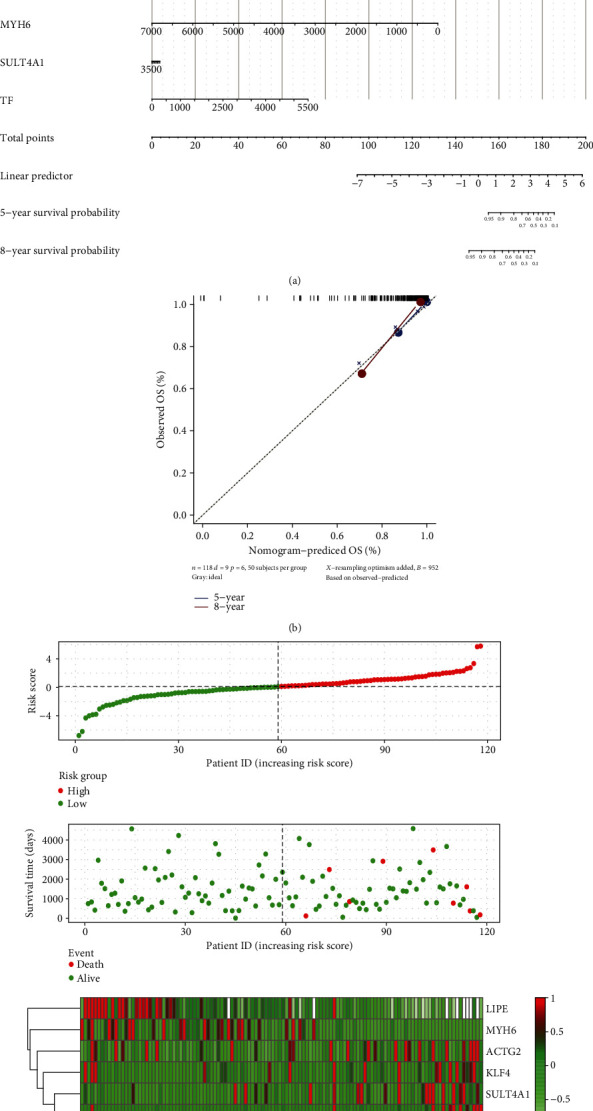
The expression of key genes affects the prognosis of patients with thymoma. (a) Nomogram for predicting overall survival in patients with thymoma. (b) Plots depict the calibration of each model in terms of agreement between predicted and observed 5-year and 8-year outcomes. (c) Risk factor correlation diagram. The green dot was the survival thymoma patient, and the red dot was the dead thymoma patient. The dotted line was the median risk score, the left side was the low-risk group, and the right side was the high-risk group.

Multivariate survival analysis was performed by Cox regression model, and nomogram was generated by Cox regression coefficients. The nomogram showed that low expression of LIPE was a risk factor for predicting the overall survival time of thymoma at 5 and 8 years (Figure 4(a)). Calibration plots showed that the nomograms performed well compared with an ideal model (Figure 4(b)). In addition, Cox risk ratio model suggested that the survival rate of the high-risk population for thymoma was poor (Figure 4(c)). Among them, low expression of LIPE and MYH6 and high expression of ACTG2, KLF4, SULT4A1, and TF were important risk factors.

### 3.5. Changes of Immune Microenvironment in Thymoma

**Figure 5:**
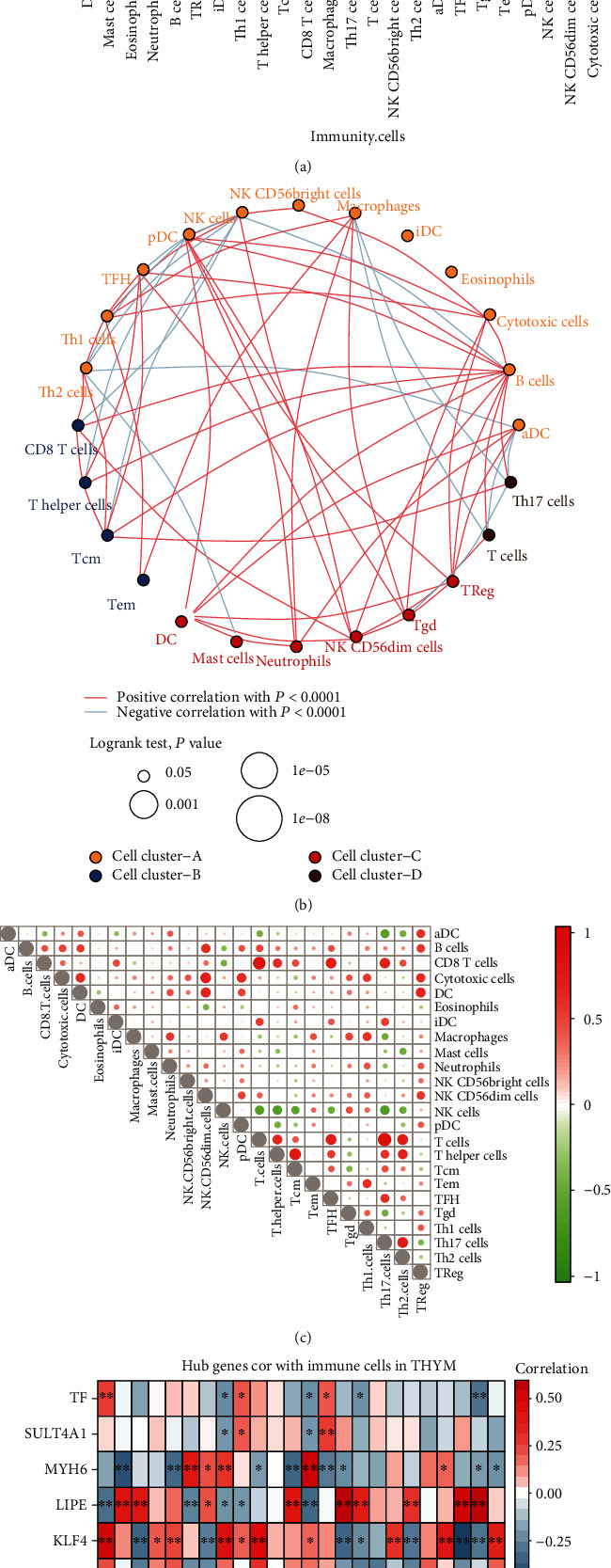
Immune cell infiltration in thymoma. (a) The difference of immune cell infiltration between thymoma and control. The blue line represents a significant difference. (b) Clustering of immunocytes with differential infiltration. The red line represents the positive correlation between immune cells, and the blue line represents the negative correlation. (c) Correlation between immune cells in thymoma. Red represents positive correlation between immune cells, and blue line represents negative correlation. The size of the node represents the size of the correlation coefficient. (d) Correlation between key genes and immune cells. Red represents positive correlation between immune cells, and blue line represents negative correlation. ^∗^*P* < 0.05 and ^∗∗^*P* < 0.01.

By comparing the immune cell infiltration between thymoma and control, we found that dendritic cells (DC) decreased most significantly in thymoma (Figure 5(a)). These differentially infiltrated immune cells were clustered into four groups (Figure 5(b)). The strongest correlation was found between T cells and CD8 T cells or Th17 cells in thymoma tissues (Figure 5(c)). In addition, we analyzed the correlation between key genes and immune cells (Figure 5(d)). LIPE had the strongest positive correlation with T cells and Th2 cells, MYH6 had the strongest positive correlation with NK cells, TF, KLF4, and aDC had the strongest positive correlation, SULT4A1 and pDC had the strongest positive correlation, and ACTG2 and neutrophils had the strongest positive correlation.

### 3.6. Regulatory Factors Associated with Thymoma

**Figure 6:**
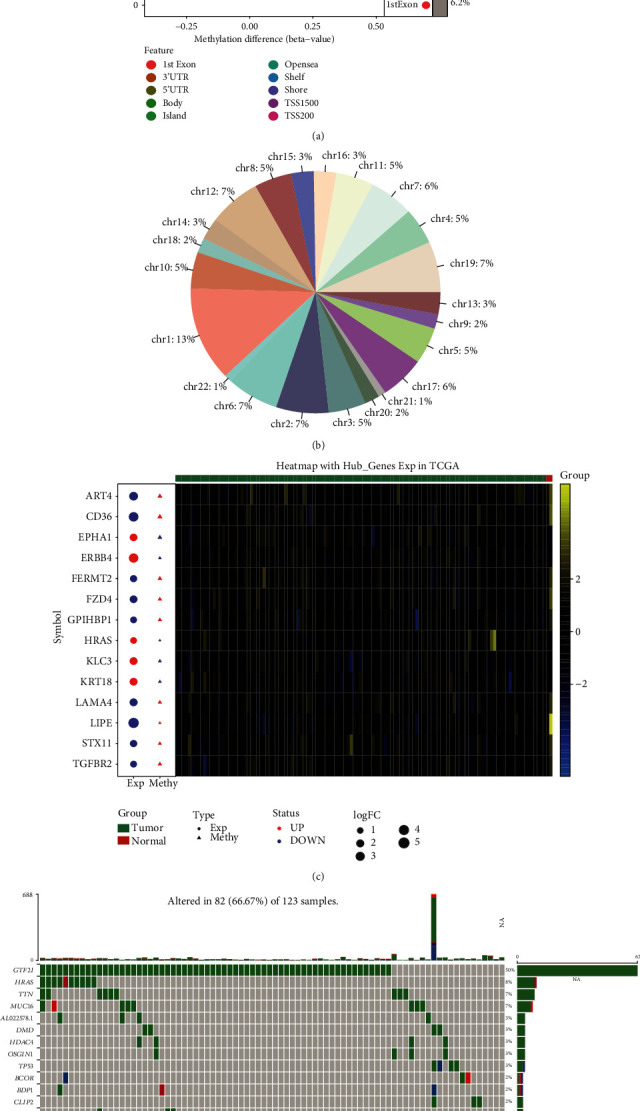
Methylation and mutation in thymoma. (a) Differential methylation sites between thymoma and control. (b) The proportion of methylation sites in different chromosomes. (c) The expression and methylation of methylation factors. Red node represents upregulation, and blue node represents downregulation. Yellow represents positive gene expression, while blue represents negative gene expression. (d) The top 20 genes with the highest mutation frequency in thymoma. Each cell represents a sample.

By comparing gene methylation modifications between thymoma and control, we obtained 943 differential methylation sites (Figure 6(a)). Among them, the methylation sites of chr1 accounted for the most, accounting for 13% (Figure 6(b)). Fourteen genes were identified as methylation factors because they had opposite levels of methylation and expression (Figure 6(c)). Among them, LIPE was significantly associated with OS in thymoma. In addition, GTF2I, the gene with the highest frequency of mutations in thymoma, was missense mutation in all samples (Figure 6(d)).

## 4. Discussion

Like other malignant tumors, the growth and proliferation of thymoma have many biological factors. However, the exact molecular basis of thymoma occurrence remains unclear. In this study, the possible molecular mechanism and regulatory factors of thymoma were explored through multiomics.

Early studies have shown that changes in certain genes seem to be associated with the development of thymic tumors [20, 21]. Our data suggest that there is a large difference in gene expression between thymoma and control. By identifying coexpression network constructed by genes with larger variance, module genes with high correlation with thymoma were obtained. Intersection with differentially expressed genes yielded 913 genes possibly associated with thymoma development.

GO functional enrichment analysis is very powerful and widely used to identify biological functions of gene expression data [22]. In the GO functional enrichment results, MAPK cascade, ERK1 and ERK2 cascade, response to transforming growth factor beta (TGF-*β*), and Wnt signaling pathway were mainly involved. Mitogen-activated protein kinase (MAPK) is a complex and interrelated signal cascade, which is closely related to the occurrence and progress of tumor, and plays an important regulatory role in cell proliferation, differentiation, migration, and survival [23, 24]. ERK 1/2 is also an effective target for anticancer [25]. Studies have shown that MAPK signal and ERK 1/2 were significantly activated in thymoma [26]. TGF-*β* inhibited apoptosis and had reduced expression of IFN-*γ* in effector cell, a key mediator of antitumor immunity [27]. Recently, it had been proved that Wnt pathway was activated in human thymoma, which may be involved in the tumorigenesis [28]. These findings further confirmed that a variety of inflammatory processes and cytokines were involved in the pathogenesis of thymoma.

In addition, in KEGG enrichment results, ECM also regulated intercellular communication, cell connectivity plasticity, and cell adhesion molecules interacting with various cytokines/chemokines or growth factors [29, 30]. There were 34% of the genes in the ECM-receptor interaction pathway mutated repeatedly in cancer [31]. Focal adhesion kinase (FAK) is highly expressed in thymic epithelial tumors and can be used as an independent prognostic biomarker [32]. PPAR*γ* overexpression more than doubled insulin-stimulated thymoma viral protooncogene phosphorylation during low lipid availability [33]. GSEA results had the same terms as KEGG enrichment results, in which cholesterol accumulation was a common feature of cancer tissues. Recent evidence showed that cholesterol played a crucial role in the progress of cancer including breast, prostate, and colorectal cancer [34]. Activation of cGMP PKG signal may promote the growth of cervical cancer cells [35].

By screening the DEGs that had a significant impact on the prognosis of thymoma, we identified LIPE, MYH6, ACTG2, KLF4, SULT4A1, and TF as key genes. LIPE was also predicted as a new prognostic marker of thymoma in other studies [36]. Consistent with our analysis, MYH6 was differentially expressed in thymoma [37]. We found that MYH6 may be a potential target for thymoma. ACTG2, KLF4, SULT4A1, and TF were all involved in the occurrence or development of cancer, but their biological significance in thymoma was not clear [38–41]. This needs further study and discussion of the follow-up experiments.

From the perspective of immune microenvironment, innate immune cells such as DC and adaptive immune cells such as T cells played an important role in thymoma [13, 42, 43]. There was a strong correlation between LIPE and immune cells, suggesting that LIPE may participate in the prognosis of thymoma by regulating the immunity system. Interestingly, we found that LIPE was also a gene regulated by methylation. DNA and RNA methylation genes are commonly studied as biomarkers [44, 45], which also seems to be a way for LIPE to participate in the development of thymoma [46]. On the other hand, genetic difference in thymoma was also an effective way to screen potential therapeutic targets [9]. GTF2I mutation occurs at high frequency in thymoma and is a marker of good prognosis [47].

However, this study also had some limitations. Firstly, conclusions may be limited by small samples, especially control samples. Secondly, the results of this study had not been verified by molecular experiments, so the interpretation of the results may be cautious. In this study, the possible molecular changes and pathogenesis of thymoma were investigated using the multiomics data from TCGA database. This study identified key genes related to the prognosis of thymoma, including LIPE, MYH6, ACTG2, KLF4, SULT4A1, and TF. The expression of these genes in thymoma may be a promising biomarker, which needs further study.

## 5. Conclusion

In this study, potential targets associated with thymoma were identified by combining thymoma-related gene expression, methylation, and mutation data. Using a variety of bioinformatics analysis methods, we found that important genes related to thymoma were associated with immune inflammatory response. LIPE, MYH6, ACTG2, KLF4, SULT4A1, and TF were the key genes affecting the prognosis of thymoma. Among them, LIPE was also modified by methylation.

## Data Availability

Thymoma mRNA-seq expression data, methylation data, mutation data, and clinical materials were obtained from TCGA website (https://portal.gdc.cancer.gov/).
